# Early perfusion changes in patients with recurrent high-grade brain tumor treated with Bevacizumab: preliminary results by a quantitative evaluation

**DOI:** 10.1186/1756-9966-31-33

**Published:** 2012-04-11

**Authors:** Antonello Vidiri, Andrea Pace, Alessandra Fabi, Marta Maschio, Gaetano Marco Latagliata, Vincenzo Anelli, Francesca Piludu, Carmine Maria Carapella, Giuseppe Giovinazzo, Simona Marzi

**Affiliations:** 1 Radiology and Diagnostic Imaging Department, Regina Elena Cancer Institute, Via Elio Chianesi 53, 00144, Rome, Italy; 2 Neurology Division, Regina Elena Cancer Institute, Rome, Italy; 3 Oncology Department, Regina Elena Cancer Institute, Rome, Italy; 4 Department of Bioimaging and Radiological Sciences, Institute of Radiology, A. Gemelli Hospital 53, Rome, Italy; 5 Neurosurgery Department, Regina Elena Cancer Institute, Rome, Italy; 6 Radiation Oncology Department, Regina Elena Cancer Institute, Rome, Italy; 7 Medical Physics Laboratory, Regina Elena Cancer Institute, Rome, Italy

**Keywords:** Perfusion CT, Anti-angiogenic therapy, Bevacizumab, Brain tumor, Hypoxia

## Abstract

**Background:**

To determine whether early monitoring of the effects of bevacizumab in patients with recurrent high-grade gliomas, by a Perfusion Computed Tomography (PCT), may be a predictor of the response to treatment assessed through conventional MRI follow-up.

**Methods:**

Sixteen patients were enrolled in the present study. For each patient, two PCT examinations, before and after the first dose of bevacizumab, were acquired. Areas of abnormal Cerebral Blood Volume (CBV) were manually defined on the CBV maps, using co-registered T1- weighted images, acquired before treatment, as a guide to the tumor location. Different perfusion metrics were derived from the histogram analysis of the normalized CBV (nCBV) maps; both hyper and hypo-perfused sub-volumes were quantified in the lesion, including tumor necrosis. A two-tailed Wilcoxon test was used to establish the significance of changes in the different perfusion metrics, observed at baseline and during treatment. The relationships between changes in perfusion and morphological MRI modifications at first follow-up were investigated.

**Results:**

Significant reductions in mean and median nCBV were detected throughout the entire patient population, after only a single dose of bevacizumab. The nCBV histogram modifications indicated the *normalization* effect of bevacizumab on the tumor abnormal vasculature. An improvement in hypoxia after a single dose of bevacizumab was predictive of a greater reduction in T1-weighted contrast-enhanced volumes at first follow-up.

**Conclusions:**

These preliminary results show that a quantification of changes in necrotic intra-tumoral regions could be proposed as a potential imaging biomarker of tumor response to anti-VEGF therapies.

## Background

Despite new treatments, the median survival of Malignant Gliomas (MGs) remains poor, ranging from 12 to 15 months for Glioblastoma Multiforme (GBM) and from 2 to 5 years for anaplastic gliomas. Such a dismal prognosis can mainly be ascribed to the rapid onset of radio and/or chemo-resistance, as well as to the limited therapeutic options available for MGs which recur after standard treatment [1-3].

Glioblastoma Multiforme (GBM) is a highly vascular brain tumor with an elevated expression of Vascular Endothelial Growth Factor (VEGF), a protein that promotes endothelial cell proliferation and migration and, consequently, tumor angiogenesis. Bevacizumab, a humanized monoclonal antibody that inhibits VEGF, administered alone or combined with cytotoxic agents, has shown promising results in terms of outcome of disease treatment in progressive MGs [4-6].

Standard criteria to determine the response to treatment are based on the evaluation of morphological Magnetic Resonance Imaging (MRI), that allows dimensional measurements of both contrast-enhancing and non-enhancing components (infiltration component), depicted on post-contrast T1-weighted and T2-weighted/Fluid–Attenuated Inversion Recovery (FLAIR) sequences, respectively [7].

Non-morphological techniques, such as Perfusion Computed Tomography (PCT) or Perfusion MRI [8-12], diffusion and spectroscopic MRI [13-15] have been proposed as tools that may offer more detailed information about the outcome of anti-angiogenic therapies. Of all the diagnostic modalities available, PCT imaging appears to be an appropriate and powerful not-invasive technique to measure the hemodynamic properties of tissues, such as blood volume, vessel leakiness and permeability [8].

The purpose of the present study was the early monitoring of the effects of bevacizumab in patients with a recurrent high-grade glioma, with a PCT examination before and after the first dose of the drug. We hypothesized that a quantitative evaluation of the changes in tumor perfusion during treatment could be predictive of the response to the anti-angiogenic therapy.

## Methods

### Patient population and study design

This prospective, single-center, open-label trial was approved by our Ethic Committee and informed consent was obtained from each patient before the study. From June 2009 to May 2011, a total of 25 patients met the following selection criteria and were prospectively enrolled in the study. Patients were eligible for the study if they had: *(i)* a pathologically proven high-grade malignant glioma (anaplastic astrocytoma, anaplastic oligoastrocytoma, anaplastic oligodendroglioma, or GBM); *(ii)* undergone surgery; (*iii*) a recurrent or progressive disease after chemo-radiotherapy (after a total dose of 60 Gy, 2 Gy per fraction, with concurrent and/or sequential Temozolomide); (i*v*) a Karnofsky performance status (KPS) greater than 60; and (*v*) if they were at least 18 years old. Among 25 patients who met the selection criteria, 9 were excluded from the analyses for inadequate PCT examination (3 patients), lack of the second PCT exam for a rapidly deteriorating condition (4 patients) or lost from follow-up (2 patients).

The final study cohort included 16 patients, 6 female and 10 male with an average age of 47.6 years (range, 34–67 years). Patient and tumor characteristics are summarized in Table 1. Patients received bevacizumab as a monotherapy or combined with other therapies, (Table 1). Patients also received corticosteroids as clinically demanded. Bevacizumab was administered every 3 weeks with a dose of 15 mg/Kg, until disease progression, refusal of patient or intolerable toxicity. The Progression Free Survival (PFS) was estimated from the beginning of anti-angiogenic therapy to radiologic and/or neurological progression. The overall survival (OS) was defined from the beginning of anti-angiogenic therapy to death.

**Table 1:** Patient, tumor characteristics and outcome of Bevacizumab

**Patient n°**	**Sex**	**Age**	**Location**	**Initial Diagnosis**		**Before Treatment**	**Other concurrent Therapies**	**RANO Response at 1° follow-up**	**PFS**
				**Histology**	**KPS**	**KPS**			
1	F	65	R P	GBM	70	70	FTM	Partial	No progress
2	M	34	L T	AOA	80	90	-	Stable	1.3
3	M	67	R F T	GBM	90	70	FTM	Stable	4.5
4	M	27	R T P	AOD	100	80	FTM	Stable	5.0
5	M	49	L F	AOD	100	70	TMZ	Stable	2.1
6	M	41	L F	AOA	100	70	TMZ	Stable	3.1
7	M	62	L T	GBM	100	80	FTM	Stable	4.0
8	F	42	L T	AA	70	70	FTM	Stable	3.0
9	F	41	R T	GBM	80	70	TMZ	Stable	No progress
10	M	50	L T P	GBM	80	80	TMZ + FTM	Progression	2.2
11	M	60	L F P	GBM	90	90	FTM	Progression	1.6
12	M	43	CC	GBM	100	80	-	Partial	2.9
13	F	48	R T P	GBM	70	80	-	Progression	2.0
14	F	43	L T P	GBM	80	80	FTM	Partial	No progress
15	F	42	L T	AOD	100	80	-	Partial	No progress
16	M	48	L P	AOD	100	80	-	Partial	4.0

Abbreviations: Sex: *M,* male; *F*, female. Location: *R*, right; *L*, left; *P*, parietal; *T*, temporal; *F*, frontal; *CC*, corpus callosum. Histology: *GBM,* glioblastoma multiforme; *AOA*, anaplastic oligoastrocytoma; *AOD*, anapalstic oligodendroglioma; *AA*, anaplastic astrocytoma; *KPS*, Karnofsky performance status at initial diagnosis and before treatment with bevacizumab. *FTM*, fotemustine; *TMZ*, temozolamide. *PFS*, progression free survival counted from the onset of treatment with bevacizumab to radiological and/or neurological progression as months.

For each patient, a baseline PCT was performed before the onset of treatment and the first dose of bevacizumab was administered the same day. The second PCT was performed immediately before the second dose of bevacizumab, with a median interval of 3 weeks (range, 2.8–3.6 weeks) from the onset of treatment. All patients underwent a baseline MRI exam within two weeks before the onset of treatment and a second MRI exam after the third dose of bevacizumab, with a median interval of 8.7 weeks, (range, 8.5 – 13 weeks) from the start of treatment.

### Conventional MR imaging: acquisition and volume quantification

MRI was performed in the first 10 patients with a 0.5 T superconductive system (Gyroscan, Philips Healthcare, Eindhoven, The Netherlands) and in the remaining 6 patients with a 1.5 T superconductive system (Optima^TM^ MR450w, GE Medical System, Waukesha, WI), using a standard birdcage head-coil and a 16-channel phased array head-coil, respectively. Because it was recognized that contrast-enhancement is nonspecific and patients treated with anti-angiogenic agents may develop tumor recurrence characterized by an augmented non-enhancing component [16], both FLAIR and contrast-enhanced T1-weighted sequences were considered for the response assessment to treatment [7]. On the 0.5 T system, axial FLAIR images were obtained with the following parameters: TI = 2000 ms, TE/TR = 150 ms/6000 ms, slice thickness = 6 mm; matrix size = 512 × 512 and voxel size = 0.5 × 0.5 × 6.0 mm^3^. Contrast-enhanced T1-weighted spin-echo (SE) images were acquired on multiple planes (axial, coronal and sagittal) after the administration of Gadopentate Dimeglumine (Gd-DTPA, Magnevist, Bayern Shering Pharma AG, Berlin, Germany) at 0,2 mmol per kilogram of body weight (TR/TE = 15 ms/355 ms, slice thickness = 6 mm; matrix size = 512 × 512 and voxel size = 0.5 × 0.5 × 6.0 mm^3^). On the 1.5 T system, FLAIR images were obtained with the following parameters: TI = 2750 ms, TE/TR = 144 ms/11000 ms, slice thickness = 4 mm; matrix size = 512 × 512 and voxel size = 0.5 × 0.5 × 4.0 mm^3^. Contrast-enhanced T1-weighted SE sequences were obtained after the administration of 0.2 mmol/Kg of Gd-DTPA, with TR/TE = 20 ms/460 ms, and the same spatial resolution parameters indicated above.

Volumes of signal abnormality on both axial FLAIR and contrast-enhanced T1-weighted images (V_FLAIR_ and V_T1_), pre-treatment and at the first follow-up, were segmented using a semi-automated region growing algorithm with 3D Slicer Software [17]. All defined volumes of interest (VOIs) excluded resection cavities and special attention was paid to consistency of tumor and edema delineations between the two MRI scans.

### CT perfusion imaging

PCT examinations were performed by using a 128-section (Brilliance CT 128-slice CT system- Philips Medical Systems, Eindhoven, Holland) multidetector-row computed tomography scanner. A preliminary un-enhanced CT scan was obtained to localize the tumor at a slice thickness of 5 mm. Fifty milliliters of nonionic iodinated contrast medium (iopamidol-370 mg I/mL, Bracco, Milan, Italy) was injected at a rate of 5 mL/s through the antecubital vein. Five seconds after the injection began, a 60 s cine scan with 2 s interval was acquired at the chosen slice locations. Eight 5-mm-thick axial sections were acquired resulting in a total coverage of 4 cm. Particular attention was paid to investigate the same portion of brain volume before and during treatment for each patient, assuring that the head and neck were relaxed but without rotation in either plane.

The dose per scan was calculated by ImPACT CT Patient Dosimetry Calculator (v. 0.99×, Medical Devices Agency, London), resulting in a total effective dose less than 5 mSv.

CT acquired images were sent to a commercially available workstation (Brain Perfusion, Brilliance Workspace Portal, v. 2.5.1.15, Philips Medical Solutions, Eindhoven, Holland) to generate perfusion maps. A neuroradiologist (*blinded to the review process*) selected the Anterior Cerebral Artery (ACA) or alternatively the Middle Cerebral Artery (MCA) as input artery; a large venous structure, such as the sagittal sinus was chosen as the input vein. To avoid partial volume effects the reference vessels had to be well recognizable, large enough and sufficiently orthogonal to the scan section. Parametric Cerebral Blood Volume (CBV) maps were then generated and stored.

### Volume of interest definition on the CBV maps

**Figure 1:**
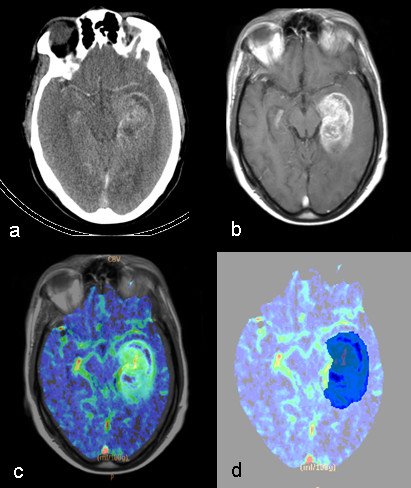
**Volume of Interest delineation.** Axial CT slice illustrating a section of the tumor (**a**); transverse contrast-enhanced T1-weighted image co-registered to the CT slice (**b**); co-registered transverse contrast-enhanced T1-weighted image overlaid on the CBV map (**c**); the user-defined region of abnormal perfusion on the CBV map (in blu) (**d**).

For each patient, pre-treatment contrast-enhanced T1-weighted images were accurately co-registered with the two PCT studies, using the rigid body transformation module of 3D Slicer Software, based on the mutual information algorithm. Before delineating the VOI on the CBV maps, a visual inspection was performed to ensure an adequate alignment between MR/CT studies. CBV maps were then overlaid on the co-registered T1-weighted images that were used to guide the tumor location. An expert radiologist was asked to manually identify the abnormal CBV areas (necrotic as well as hyper-perfused), on the eight slices acquired. Taking the contralateral hemisphere as control region, a visual inspection was performed to verify whether arterial or venous structures were involuntary included inside the VOI (an example is illustrated in Figure 1).

### Quantitative analysis of the CBV maps

The quantitative analysis of the perfusion maps was performed using the Matlab code (Release 7.4.0, The Mathworks Inc., Natick, Massachusetts). A script was developed by a medical physicist (*blinded to the review process*), with more than 10 years’ experience in data analysis, to perform calculations based on voxel-by-voxel information. The CBV maps, generated by the commercial workstation, were loaded in the Matlab workspace and divided by the CBV mean inside a healthy region of about 1 cm^2^, in the hemisphere contralateral with respect to the lesion, to obtain the *normalized* CBV (nCBV) maps. For each patient, the same region was chosen to derive the nCBV maps at baseline and after the first dose of bevacizumab. Assuming a fixed nCBV bin size of 0.5, the distribution of the voxel counts as a function of the bin locations (differential histogram) was recorded and displayed for each PCT.

The VOIs with abnormal CBV delineated by 3D Slicer Software (Figure 1) were loaded in the Matlab workspace and used to quantify, within them, the distribution of nCBV values (nCBV histogram). Specific hypo- and hyper-perfused sub-volumes were calculated, as the absolute voxel count within the VOIs in which nCBV values were less or greater than fixed thresholds, respectively. Three hypo-perfused sub-volumes were determined as the volumes with nCBV less or equal to 1.0, 0.5 and 0 (tumor necrosis), defined as V_≤ 1.0,_ V_≤ 0.5_ and V_= 0_. Analogously, five hyper-perfused sub-volumes were determined as the volumes with nCBV more or equal to 1.5, 2.0, 2.5, 3.0, and 3.5 defined as V_≥ 1.5_-V_≥ 3.5_.

### Statistics

A two-tailed Wilcoxon test for paired samples was used to establish if changes of the same variable, observed at different time points, were significant. The relationships between modifications based on perfusion metrics and morphological MRI changes/PFS/OS were investigated using the Pearson correlation test. Unless otherwise indicated, summary statistics were reported as median and standard deviations. A two-sided p-value ≪ 0.05 was considered to indicate statistical significance. The MedCalc software (Version 9, Mariakerke, Belgium) was used for the statistical analyses.

## Results

According to RANO criteria, five patients showed a partial response, eight were described as clinically stable and three had a progression of disease (Table 1). From June 2009 up to now, all but 4 had a progression and died of progressive disease. The median PFS for the remaining 12 patients was 3.0 months (range, 1.3–5.0 months), with a median OS of 4.8 months (range, 1.6–14.8 months).

### T1 post-contrast and flair volumetric analysis

Before treatment, the volumes V_T1_ and V_FLAIR_ were 27.4 ± 13.4 cm^3^ and 111.7 ± 53.0 cm^3^, respectively and at the first follow-up, were 16.1 ± 33.8 cm^3^ and 112.8 ± 80.9.0 cm^3^, respectively. As percentages, V_T1_ and V_FLAIR_ at the first follow-up relative to the initial volumes, were 59.2 ± 88.3% and 97.1 ± 70.2%, respectively, showing a decrease in V_T1_ and a stability of V_FLAIR_. Considering all patients, no statistical significance appeared in either of the sequences, both in absolute units and percentages.

### Analysis of changes in CBV

The nCBV mean, median and standard deviation (SD) within the VOI showed a strongly significant decrease during treatment, throughout the entire patient population (Table 2): the baseline values were 2.3, 2.5 and 1.6, respectively, while after the first dose of bevacizumab they were 1.2, 1.5 and 1.0, respectively. Changes in mean and median nCBV reflect an appreciable tumor vasculature normalization because of the effect of the anti-angiogenic agent.

**Table 2:** Results of Wilcoxon test, to establish if early changes of perfusion metrics are significant

**Summary statistics for nCBV**	**Mean**	**Median**	**SD**		
*p* value	0.0006	0.0042	0.0076		
Hypo-perfused sub-volumes	V_≤ 1.0_	V_≤ 0.5_	V_= 0_		
*p* value	0.43	0.78	0.90		
Hyper-perfused sub-volumes	V_≥ 1.5_	V_≥ 2.0_	V_≥ 2.5_	V_≥ 3.0_	V_≥ 3.5_
*p* value	0.0001	0.0001	≪0.0001	≪0.0001	≪0.0001

Abbreviations: *nCBV* = *normalized* cerebral blood volume; *SD* = standard deviation; *V*_*≤ 1.0*_ = is the total number of voxels, within the volume of interest, in which nCBV is ≤ 1.0 (analogously for V_≤ 0.5_ and V_= 0_); *V*_*≥ 1.5*_ = is the total number of voxels, within the volume of interest, in which nCBV is ≥ 1.5 (analogously for V_≥ 2.0_-V_≥ 3.5_).

All the hyper-perfused sub-volumes (V_≥ 1.5_–V_≥ 3.5_) showed an even more significant decrease during treatment, with p values ≤ 0.0001. On the contrary, the changes of the hypo-perfused sub-volumes, including the necrotic region (V_=0_), were not significant (Table 2).

**Figure 2:**
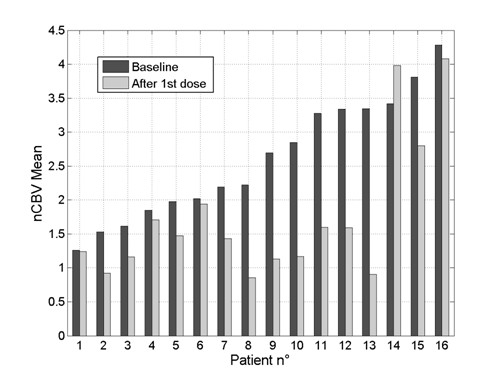
**Normalized cerebral blood volume for each patient.** Mean values of the *normalized* cerebral blood volume (nCBV), before treatment and after the first dose of bevacizumab, for each patient.

The nCBV mean values inside the VOI, before treatment and after a single dose of bevacizumab, are displayed for each patient in Figure 2. Baseline values have been expressly sorted in ascending order to understand whether the *normalization* effect of bevacizumab could somehow depend on the perfusion level of the lesion before treatment.

### Correlations between early CBV changes and MRI response/PFS/OS

Only the percentage change of the necrotic sub-volume (V_=0_), relative to the pre-treatment value, showed a significant relationship with the percentage V_T1_ modification at the first follow-up (correlation coefficient *r* = 0.829, 95% Confidence Interval = 0.551–0.942, *p*-value = 0.0001). None of the variations of the other parameters, including nCBV mean, median, SD or any of the hyper-perfused sub-volumes, showed significant relationships with V_T1_ and V_FLAIR_ changes.

A tendency of correlation was found between the percentage change of V_=0_ and PFS (*p* = 0.09), while no correlation emerged between the observed perfusion changes and OS.

In the subgroup of patients stable or with a progression of disease (11 in total), the mean changes of V_≤ 1.0,_ V_≤ 0.5_, V_= 0_ were 61.5%, 68% and 4.3%, respectively; while in the subgroup of patients with partial response (5 in total), the changes were 10.4%, -9.4% and -59.1%, respectively. Analogously, for patients stable or in progression, the variations of V_≥ 1.5_, V_≥ 2.0,_ V_≥ 2.5,_ V_≥ 3.0,_ V_≥ 3.5_ were −44.1%, -61.8%, -51.2%, -51,7%, -60.2%_,_ respectively, while for partially responding patients, they were −53.1%, -65.2%, -70.%, -75.5%, -81.4%, respectively.

### Representative cases

#### Case 1

**Figure 3:**
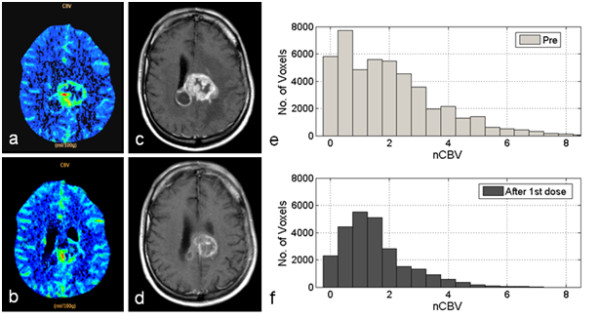
**Representative case 1.** A 43-year-old man (Patient 12) affected by a glioblastoma multiforme in the corpus callosum. Cerebral Blood Volume (CBV) map illustrating a section of the lesion before treatment (**a**); co-registered transverse post-Gd T1-weighted image showing an area of increased contrast enhancement, before treatment (**b**); CBV map acquired during treatment indicates a decreased blood volume in the region of interest (**c**); transverse post-Gd T1-weighted image, acquired 7 weeks after the onset of treatment, shows a decrease in contrast enhancement (**d**). Differential histogram of *normalized* CBV (nCBV) values inside the volume of interest, before treatment (**e**) and after a single dose of bevacizumab (**f**), showing a decrease in both hyper/hypo-perfused subvolumes.

In Figure 3 the case of a 43-year-old man affected by GBM in the corpus callosum is illustrated (Patient 12), who received bevacizumab as single therapy. Comparing the CBV maps, acquired before and during treatment, a decreased blood volume is noticeable in the region of interest; this behavior is more exhaustively illustrated by a comparison between the nCBV histograms within the entire volume investigated by the PCT. The two distributions of the nCBV values indicate a reduction in both hyper-/hypo-perfused sub-volumes, in accordance with a decreased hyperintensity, shown by the post-constrast T1-weighted and FLAIR (data not shown) images, acquired 7 weeks after the onset of treatment. The patient was classified as partially responding, in accordance with RANO criteria. Approximately 1 month after the MRI scan, the patient showed a rapid deterioration of the clinical condition due to meningitis and died approximately 1 month later.

#### Case 2

**Figure 4:**
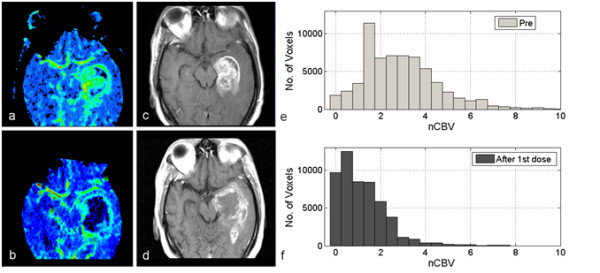
**Representative case 2.** A 50-year-old man affected by a glioblastoma multiforme in the left temporal region (Patient 10): Cerebral Blood Volume (CBV) map illustrating a section of the lesion before treatment (**a**); co-registered transverse post-Gd T1-weighted image showing the area of increased contrast enhancement, before treatment (**b**); *normalized* CBV (nCBV) map showing the modification of the blood volume after a single dose of bevacizumab (**c**); co-registered transverse post-Gd T1-weighted image acquired at the first follow-up, showing an augmented area of contrast enhancement and necrosis (**d**). Differential nCBV histogram inside the volume of interest, before treatment (**e**) and after a single dose of bevacizumab (**f**), showing a decrease in the hyper-perfused regions but an increase in the hypo-perfused sub-volumes.

Figure 4 shows a 50-year-old man affected by a GBM in the left temporal region (Patient 10), who received bevacizumab with concurrent temozolamide and fotemustine. The CBV map, acquired after the first dose of bevacizumab, shows rapid growth of the necrotic area, that has completely replaced the hypervascularized region documented by the baseline CBV map. This is displayed in more detail by the nCBV histograms, showing a significant decrease in the hyper-perfused regions but, contemporary, a marked increase in the hypo-perfused sub-volumes inside the VOI, in particular V_=0_ increases by 425% with respect to the baseline value. These abnormal CBV areas seem to be predictive of the subsequent changes in contrast enhancement, as documented by the post-Gd T1-weighted images acquired before (Figure 4c) and at 10 weeks from the onset of treatment (Figure 4d). The patient was defined as progressive and died two months after the MRI scan.

## Discussion

In the present study, we aimed at investigating whether PCT may be used to obtain early non-invasive imaging biomarkers of the response to anti-angiogenic therapy, in patients affected by recurrent high-grade gliomas. There is strong interest in validating biomarkers which could prove to be predictive of response to treatment, to better stratify the patients most likely to benefit from these therapies.

Our results indicate that large reductions in mean and median nCBV can be detected throughout the entire patient population, after only a single dose of bevacizumab. From the analysis of each patient (Figure 2), it is noticeable that mean nCBV after bevacizumab has a tendency to approach the value of 1, that represents the mean nCBV of the normal appearing brain tissue. The SD also significantly decreased after the first cycle of bevacizumab, indicating a narrower distribution of nCBV values within the lesion, in accordance with a reduction of the tumor vascular heterogeneity as visually documented by perfusion maps acquired during treatment. However, for an initial mean nCBV greater than 2.5, this normalization effect seems to be less efficient, suggesting that a high perfusion at baseline may correspond to reduced activity of the anti-angiogenic agent, even if this trend should be supported by further investigation on a larger patient population.

Among all different perfusion metrics derived from CBV maps, the most significant changes were found for the hyper-perfused sub-volumes (particularly *p* ≪ 0.0001 for a cutoff of nCBV greater than 2.5, Table 2). A similar method of quantitative analysis was performed by Sawlani *et al.*[11], who calculated size, mean relative CBV, mean leakage coefficient and hyperperfusion volume (HPV), in 16 patients with recurrent GBM receiving bevacizumab, both at baseline and at the first follow-up (6 weeks). The HPV, with a cutoff of relative CBV greater than 1, proved to be the metric with a significantly better correlation with the time to progression, thus it was proposed as a valid measure of response to anti-angiogenic chemotherapy. A direct comparison between the two studies is not possible, primarily because of the different timing of the perfusion studies (patients of our study underwent a perfusion exam at a median interval of 3 weeks from the onset of treatment vs 6 weeks) and, secondly, because of the different perfusion imaging modality (MR vesus CT). However, in accordance with Sawlani *et al.*[11], we observed that partially responding patients exhibited greater percentage changes in hyper-perfused sub-volumes than patients clinically stable or with disease progression (V_≥ 2.5_, V_≥ 3.0_ and V_≥ 3.5_ were -70.%, -75.5%, –81.4% versus -51.2%, -51,7%, -60.2%_,_ respectively for the two groups of patients).

In our opinion, the most interesting finding of the present investigation was derived from monitoring the less-oxygenated regions in the tumor. The early modifications in this region are the only ones which correlate with percentage changes in T1-weighted contrast-enhanced volumes at first follow-up (*p* = 0.0001).

The important role of intra-tumor hypoxia in anti-VEGF therapies has emerged from a few recent reports [15,18,19]. Masunaga *et al.*[18] evaluated the influence of bevacizumab on intra-tumor oxygenation status in mice, distinguishing between acute and chronic hypoxia resulting from limited perfusion and limited oxygen diffusion, respectively. The authors concluded that bevacizumab preferentially oxygenated the acutely Hypoxic Fraction (HF) rather than the chronically HF in the tumor. So, the remaining HF after anti-angiogenic treatment should preferentially be composed of a chronic hypoxia-rich cell population, whose control was found to have a significant impact on the local control of the tumor. Thus, the evidence of increased necrotic areas inside the lesion during therapy (as documented in Figure 4 for a patient described as clinically in progression of disease) should represent an early indication of treatment failure, due to the lack of local tumor control.

Hattingen *et al.*[15] investigated whether bevacizumab altered oxygen and energy metabolism and showed antitumoral effects in recurrent GBM, by using ^31^P and ^1^H MRSI and diffusion MRI, at baseline and after the first cycle of bevacizumab. They also indirectly evaluated blood oxygenation by a quantitative mapping of T2 and T2’ relaxation times, reporting that bevacizumab induces relative tumor hypoxia (T2’ decrease). However, the authors stated that this long-term hypoxia does not seem to promote more aggressive tumor growth, because no association was found between the T2’reduction and a shorter OS duration.

The promising but yet controversial effect of bevacizumab have been recently reported by Keunen *et al.*[20], whose data strongly suggest that vascular remodeling induced by anti- VEGF treatment may lead to a more hypoxic tumor microenvironment and, consequently, to enhanced tumor cell invasion into the normal brain. Studies combining imaging with molecular biomarkers will probably make a substantial contribution to a better understanding of the complex cellular mechanisms by which bevacizumab temporarily corrects the abnormal vasculature of tumors [9,19]. Anti-hypoxia inducible factor-1α (HIF−1α) have recently been shown to have a link with perfusion imaging. Yopp *et al.*[19] analyzed a group of patients with primary liver cancer treated with floxuridine and bevacizumab and found that reductions in tumor perfusion were greater in tumors expressing HIF−1α.

To our knowledge, this is the first investigation using PCT to evaluate the response to anti-angiogenic therapies in patients with brain tumors. Data on CT perfusion, as a biomarker in oncology, for the response to therapy are to date insufficient [8], in spite of the advantage of PCT for providing absolute perfusion data, thanks to the linear relationship between CT enhancement and contrast agent concentration compared to MR perfusion. Although the feasibility of PCT for routine diagnosis is mainly limited for the use of ionizing radiation, selecting a low kVp X-ray beam and optimizing the scanning protocol, i.e. image interval and scanning duration, it is possible to reduce the radiation dose to the patient to acceptable levels of total effective dose.

There are some limits to our study. The 4-cm coverage of PCT in cranio-caudal direction precluded us from investigating, in some patients, the entire tumor volume and, in these cases, only the central portion of the lesion was examined. Furthermore, two different MR systems were used to evaluate the V_T1_ and V_FLAIR_ changes, which represents a potential bias of the study because the magnetic field intensity affects the signal to noise ratio and may have an impact on the dimensional measurement of the V_T1_ and V_FLAIR_. However, this bias is attenuated by the fact that only relative measurements (volume variations expressed as percentages) were correlated with the different perfusion metrics, and the same MR system was used, before and at first follow-up, for the each patient.

Due to the low statistical power of the analyzed patient group, a few correlations were found between the observed perfusion changes and clinical endpoints, i.e. PFS and OS (only a tendency of correlation emerged between changes in V_=0_ and PFS). Additional studies are warranted, including a larger number of patients, to better investigate the relationships between the proposed perfusion metrics and clinical outcomes.

Finally, laborious data processing is needed for each patient to accurately co-register the acquired MR/CT exams, delineate all VOIs and obtain, by home-made software, a quantification of hyper-/hypo-perfused sub-volumes in the lesion. The proposed method of analysis not being included in routine measurements, our results are not easily reproducible by other research groups for further validation.

## Conclusions

In summary, our results underline the utility to quantify the variations of the entire distribution of CBV values in the tumor, by the use of metrics based on histogram analysis. We found that an improvement in hypoxia after a single dose of bevacizumab was a predictor of a greater reduction in T1-weighted contrast-enhanced volumes at first follow-up. We propose that a quantification of changes in necrotic intratumoral regions may be considered as an alternative imaging biomarker of the tumor response to anti-VEGF therapies.

## Competing interests

The authors declare that they have no competing interests.

## Authors’ contributions

All the authors have made a substantive intellectual contribution to the article. AV and SM contributed to the conception and design of the study, the analysis and interpretation of data and drafted the manuscript. AP, MM and AF contributed to the patient enrollment and helped to revise the article. VA, FP and GML helped with the coordination of the study and participated in the interpretation of data. CMC and GG participated in the design of the study and helped to revise the article.

## References

[B1] Lacroix M Abi-Said D Fourney DR Gokaslan ZL Shi W DeMonte F Lang FF McCutcheon IE Hassenbusch SJ Holland E Hess K Michael C Miller D Sawaya R A multivariate analysis of 416 patients with glioblastoma multiforme: prognosis, extent of resection, and survival J Neurosurg 2001 95 190 198 10.3171/jns.2001.95.2.0190 11780887

[B2] Stupp R Mason WP van den Bent MJ Radiotherapy plus concomitant and adjuvant temozolomide for glioblastoma N Engl J Med 2005 352 987 996 10.1056/NEJMoa043330 15758009

[B3] Park JK Hodges T Arko L Shen M Dello Iacono D McNabb A Olsen Bailey N Kreisl TN Iwamoto FM Sul J Auh S Park GE Fine HA Black PM Scale to predict survival after surgery for recurrent glioblastoma multiforme J Clin Oncol 2010 28 3838 3843 10.1200/JCO.2010.30.0582 20644085 PMC2940401

[B4] Jain RK Antiangiogenic therapy for cancer: current and emerging concepts Oncology 2005 19 7 16 Review 15934498

[B5] Vredenburgh JJ Desjardins A Herndon JE Marcello J Reardon DA Quinn JA Rich JN Sathornsumetee S Gururangan S Sampson J Wagner M Bailey L Bigner DD Friedman AH Friedman HS Bevacizumab plus irinotecan in recurrent glioblastoma multi- forme J Clin Oncol 2007 25 4722 4729 10.1200/JCO.2007.12.2440 17947719

[B6] Kreisl TN Kim L Moore K Duic P Royce C Stroud I Garren N Mackey M Butman JA Camphausen K Park J Albert PS Fine HA Phase II trial of single- agent bevacizumab followed by bevacizumab plus irinotecan at tumor progression in recurrent glioblastoma J Clin Oncol 2009 27 740 745 10.1200/JCO.2008.16.3055 19114704 PMC2645088

[B7] Wen PY Macdonald DR Reardon DA Cloughesy TF Sorensen AG Galanis E Degroot J Wick W Gilbert MR Lassman AB Tsien C Mikkelsen T Wong ET Chamberlain MC Stupp R Lamborn KR Vogelbaum MA van den Bent MJ Chang SM Updated response assessment criteria for high-grade gliomas: response assessment in neuro-oncology working group J Clin Oncol 2010 28 1963 1972 10.1200/JCO.2009.26.3541 20231676

[B8] Goh V Ng QS Miles K Computed Tomography Perfusion Imaging for Therapeutic Assessment: Has It Come of Age as a Biomarker in Oncology? Invest Radiol 2011 47 2 4 21808202 10.1097/RLI.0b013e318229ff3e

[B9] Ng CS Charnsangavej C Wei W Yao JC Perfusion CT findings in patients with metastatic carcinoid tumors undergoing bevacizumab and interferon therapy AJR Am J Roentgenol 2011 196 569 576 10.2214/AJR.10.4455 21343498

[B10] Sorensen AG Batchelor TT Zhang WT Chen PJ Yeo P Wang M Jennings D Wen PY Lahdenranta J Ancukiewicz M di Tomaso E Duda DG Jain RK A "vascular normalization index" as potential mechanistic biomarker to predict survival after a single dose of cediranibin recurrent glioblastoma patients Cancer Res 2009 69 5296 5300 10.1158/0008-5472.CAN-09-0814 19549889 PMC2824172

[B11] Sawlani RN Raizer J Horowitz SW Shin W Grimm SA Chandler JP Levy R Getch C Carroll TJ Glioblastoma: a method for predicting response to antiangiogenic chemotherapy by using MR perfusion imaging-pilot study Radiology 2010 55 622 628 20413772 10.1148/radiol.10091341 PMC2858811

[B12] Fellah S Girard N Chinot O Cozzone PJ Callot V Early evaluation of tumoral response to antiangiogenic therapy by arterial spin labeling perfusion magnetic resonance imaging and susceptibility weighted imaging in a patient with recurrent glioblastoma receiving bevacizumab J Clin Oncol 2011 10 29 308 311 10.1200/JCO.2010.32.6082 21263101

[B13] Saraswathy S Crawford FW Lamborn KR Pirzkall A Chang S Cha S Nelson SJ Evaluation of MR markers that predict survival in patients with newly diagnosed GBM prior to adjuvant therapy J Neurooncol 2009 91 69 81 10.1007/s11060-008-9685-3 18810326 PMC3022437

[B14] Nowosielski M Recheis W Goebel G Güler O Tinkhauser G Kostron H Schocke M Gotwald T Stockhammer G Hutterer M ADC histograms predict response to anti-angiogenic therapy in patients with recurrent high-grade glioma Neuroradiology 2011 53 291 302 10.1007/s00234-010-0808-0 21125399 PMC3063200

[B15] Hattingen E Jurcoane A Bähr O Rieger J Magerkurth J Anti S Steinbach JP Pilatus U Bevacizumab impairs oxidative energy metabolism and shows antitumoral effects in recurrent glioblastomas: a 31P/1H MRSI and quantitative magnetic resonance imaging study Neuro Oncol 2011 13 1349 1363 10.1093/neuonc/nor132 21890539 PMC3223092

[B16] Ellingson BM Cloughesy TF Lai A Nghiemphu PL Mischel PS Pope WB Quantitative volumetric analysis of conventional MRI response in recurrent glioblastoma treated with bevacizumab Neuro Oncol 2011 13 401 409 10.1093/neuonc/noq206 21324937 PMC3064698

[B17] Pieper S Lorensen B Schroeder W Kikinis R The NA-MIC Kit TK, VTK, pipelines, grids and 3D slicer as an open platform for the medical image computing community Proceedings of the 3rd IEEE International Symposium on Biomedical Imaging: Nano to Macro 2006 698 701

[B18] Masunaga S Liu Y Tanaka H Sakurai Y Suzuki M Kondo N Maruhashi A Ono K Reducing intratumor acute hypoxia through bevacizumabtreatment, referring to the response of quiescent tumor cells and metastatic potential Br J Radiol 2011 84 1131 1138 10.1259/bjr/38457938 21586505 PMC3473837

[B19] Yopp AC Schwartz LH Kemeny N Gultekin DH Gönen M Bamboat Z Shia J Haviland D D'Angelica MI Fong Y DeMatteo RP Allen PJ Jarnagin WR Antiangiogenic therapy for primary liver cancer: correlation of changes in dynamic contrast-enhanced magnetic resonance imaging with tissue hypoxia markers and clinical response Ann Surg Oncol 2011 18 2192 2199 10.1245/s10434-011-1570-1 21286939 PMC3137666

[B20] Keunen O Johansson M Oudin A Sanzey M Rahim SA Fack F Thorsen F Taxt T Bartos M Jirik R Miletic H Wang J Stieber D Stuhr L Moen I Rygh CB Bjerkvig R Niclou SP Anti-VEGF treatment reduces blood supply and increases tumor cell invasion in glioblastoma Proc Natl Acad Sci 2011 108 3749 3754 10.1073/pnas.1014480108 21321221 PMC3048093

